# Examining influencing factors of express delivery stations’ spatial distribution using the gradient boosting decision trees: A case study of Nanjing, China

**DOI:** 10.1371/journal.pone.0288716

**Published:** 2023-07-14

**Authors:** Qianhui He, Shijie Sun

**Affiliations:** School of Architecture, Southeast University, Nanjing, China; Jeonbuk National University, REPUBLIC OF KOREA

## Abstract

Online shopping has promoted the development of logistics and express delivery businesses. Express delivery stations are closely related to residents’ daily lives, and it is an important topic for the study of urban consumption space and commercial service space. This paper analyzed the factors influencing the spatial distribution of terminal logistics space (express delivery stations) in the process of online shopping. The gradient boosting decision trees (GBDT) was selected for analyzing the factors influencing the distribution of express delivery stations. The results demonstrated that express delivery stations’ distribution is mainly influenced by commercial retail and residential neighborhoods, showing a clustering toward consumer spaces and residential areas. This paper studied the association between express delivery stations and other functional spaces in the city, and established an analytical framework for the factors influencing the spatial distribution of express delivery stations. The research results help to improve the rationality and effectiveness of the setting and management of the terminal logistics space in the online shopping process.

## Introduction

The development of information technology has made the Internet readily available to the public and changed people’s lifestyles and shopping patterns. The traditional "face-to-face" consumption model has been altered and the relationship between consumption space and people reshaped, with both virtual cyberspace and physical space generating new features respectively. *Retail & Ecommerce Sales*, *Worldwide*, a report released by eMarketer, showed that global online retail sales have risen from 1.42 to 4.28 trillion US dollars from 2015 to 2020 [1]. Online shopping has become an important consumption method worldwide, with a growing number of consumers shopping virtually. The integration of online and offline, the export of private brands, and the diversification of commodity structure and intelligent platforms have become developing trends of online shopping.

**Fig 1:**
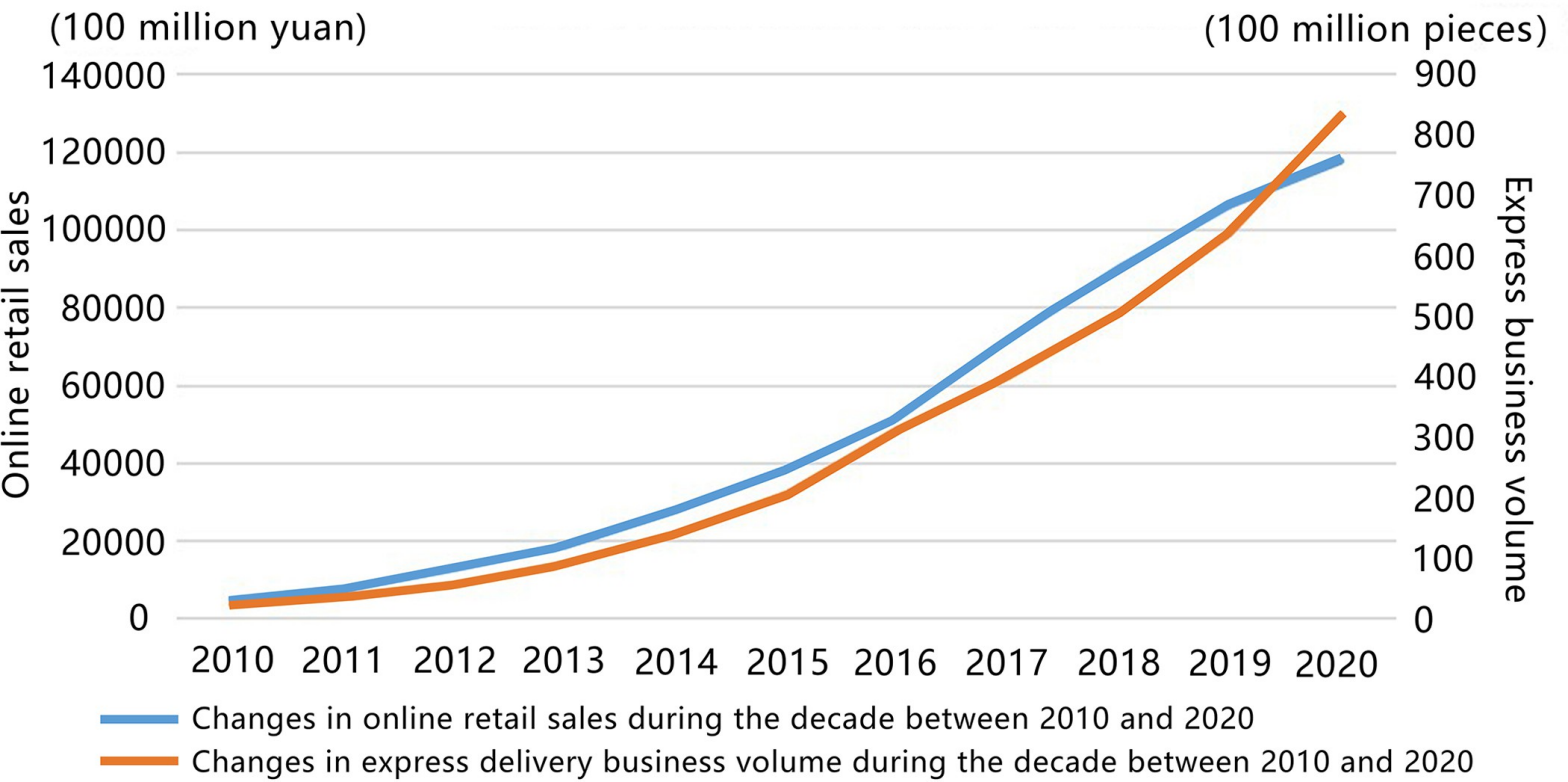
Changes in online retail sales and express delivery business volume during the decade between 2010 and 2020.

From 2010 to 2021, the proportion of e-tailing to total social retail sales in China increased from 3.2% to 24.5%. Beyond supplementing the physical business model, online shopping is now challenging the traditional model through its exuberant growth [2]. The prevalence of online shopping has promoted the development of express business [3], and the subsequent growth in China’s online shopping retail sales and express delivery businesses over the past decade have presented a higher challenge to the distribution of terminal logistics (Fig 1). With the surge in the number of express parcels, door-to-door express delivery faces problems such as insufficient manpower and mismatch of delivery time. Such a challenge has raised the concerns of Chinese government. In fact, *Several Opinions of the State Council on Promoting the Development of the Express Industry* [4] and *Guiding Opinions of the State Post Bureau on Promoting the Technological Innovation of the Postal Industry* [5] have already proposed to increase financial support and promote service intelligence to solve the "last mile" problem.

Afterwards, many e-commerce platforms and logistics companies have also started the construction of express delivery stations, including express self-pickup points, corporate express delivery stations. SF Express, Yuantong, Zhongtong, Shentong, Yunda and other express delivery enterprises have built the Cainiao network, where people can pick up their own deliveries, to provide smart supply service chains and a terminal logistics space system, while JD.com, Tmall and other e-commerce platforms have continuously improved self-built logistics, improving the quality of their delivery services as well as providing self-pickup services.

Since 2020, the Coronavirus pandemic, a major public health event, has brought about significant changes in the way society functions and the way people behave. The commercial retail industry, which is closely related to the residents, is facing major changes in purchasing ways and processes. The existing commercial service supporting system lacks the online part, which is also reflected in the configuration of express delivery stations. Currently, China’s express sites are built spontaneously under the role of the market, and the government has not intervened enough. In the current Chinese national standards, as dictated by sources such as the *Urban Residential Area Planning and Design Standards* and the *Community Commercial Facility Setup and Functional Requirements*, express delivery services have been included in the scope of regulation, but there are no specific provisions on the spatial configuration of the express delivery stations, etc. Due to the lack of scientific guidance, the development process of express delivery stations has also revealed some problems, such as lack of convenience and mismatch of resources. How to deal with the rapid development of express delivery stations has become a new challenge for city managers and planners.

In China, express delivery stations are usually considered to be distributed around service recipients, such as communities, businesses, universities, while in some other countries, it has been studied that express delivery stations may be located close to commercial facilities, public transportation facilities, etc. This paper intends to investigate which geospatial factors are associated with the distribution of express delivery stations under the role of the market, analyze the importance of these factors using gradient boosting decision tree (GBDT), and discuss the rationality and shortcomings of the spatial distribution. The findings may provide policy implications for the planning and management of this new type of commercial support facility.

## Literature review

The prevalence of online shopping has an impact on residents’ shopping behaviour [6–9], store shopping [10–13] and the layout of urban spaces in cities [14–16]. At the same time, online shopping has also promoted the development of express delivery business, with a variety of express delivery spaces popping up in the city [17]. Since 2000, self-pickup models have emerged in many countries such as the CDP (Collection and Delivery Point) in the UK [18], Pickup Point in Europe [19], and SND (Sustainable Networked Delivery) in the US [20]. In China, express delivery stations appeared in 2012 with the boom of online shopping and began to develop rapidly after 2014. In 2014, China’s express delivery entered "the era of 100 million pieces one day"[21], during which sending and receiving couriers became part of residents’ daily lives [22,23] and logistics terminals functioned as an important link of online shopping. The spatial layout of express delivery stations has become an important topic for scholars in in various countries.

The existing studies of the spatial distribution of express delivery stations are mainly based on Point of Interest (POI) data. POI is a kind of point data representing real geographic entities, usually with rich semantic features and spatio-temporal dynamic correlation characteristics, widely used in urban spatial point pattern analysis and other aspects, has become an important resource for geographers to analyse the natural geographical environment and perceive the pattern of human social activities [24,25]. Spatial analysis based on POI data: such as kernel density analysis, Getis-Ord General G analysis, Standard deviation ellipse analysis, etc. have been widely used by various scholars in the distribution of express delivery stations [26,27]. Tang and Ma (2020) studied the spatial pattern and structure of urban networked logistics in Chinese cities and found that it is characterized by hierarchical and uneven structure [26]; Li et al. (2019) studied the distribution of self-pickup points in Wuhan and found that the distribution of self-pickup points highly overlapped with residential areas and formed "central hot spots" and "edge cold spots" agglomerating in the central urban area [27]; Liu et al. (2019) found that the spatial distribution of Cainiao Post stations and China Post stations in Shenzhen presented a multi-core clustering pattern, mainly located near supermarkets, convenience stores, and the entrances of residential areas [28]. In addition, there are also some studies that proposed optimization of distance-based models for express delivery station distribution [29,30].

With the rapid growth of express delivery businesses, scholars have conducted many studies on the factors influencing the distribution of express delivery stations. Existing studies point out that population agglomeration, commercial facilities, and traffic accessibility could influence the location and distribution of express delivery stations. Existing studies find that express delivery stations tend to be distributed in populated areas. To improve the efficiency of customer delivery and pickup, express delivery stations highly rely on the distribution of customer groups [31]. Morganti et al. (2014) found that the distribution of express delivery stations in France is mostly concentrated in areas with high population density in urban centers [32]. Lemeke et al. (2016) believed that the proximity of express lockers to residential areas can reduce CO2 emissions and thereby their negative effects on the environment [33]. Yuen et al. (2018) claimed that the delivery stations ought to be located more closely to users to reduce the time cost of customers, and that express delivery stations should be set up in accordance with customers’ daily life routes [34]. Liu et al. (2019) argue that express delivery stations tend to be located close to the entrances of communities, companies, and industrial parks and are impacted by the level of economic development and population distribution [27].

Some researchers have also found a strong relationship between express delivery stations and commercial spaces. Morganti et al. (2014) found that the express delivery stations in France are concentrated in the main commercial blocks and that cooperation with other commercial operators is beneficial to both sides [35]. Milioti et al. (2020) suggested that express delivery stations can be arranged in conjunction with the daily consumption places of customers such as pharmacies [36].

Some researchers believe that the distribution of express delivery stations could be impacted by traffic conditions: Mehmood et al. (2014) argue that there is a strong correlation between express delivery stations distribution and urban street networks [37]. Liu et al. (2019) found that some groups take public transportation to express delivery stations [38]. Morganti et al. (2014) found that each train station area in France has a pick up station within 600m and concluded that express delivery station distribution is related to commuting [32].

In the existing studies, the analysis of factors influencing the spatial distribution of express delivery stations has mostly used textual analysis, mathematical and statistical analysis and comparative analysis. Liu et al. (2019) have directly compared the relationship between express delivery stations and land use and road networks, arguing that the distribution of express delivery stations is close to entrances and exits of communities, companies, etc. [28]. Morganti et al. (2014) counted the number of express delivery stations around transport stations and found that pick up stations spread around train stations [32]. This paper proposes to bring a new method to this type of research. In recent years, machine learning models have been widely used in urban research. Many scholars have used GBDT model to explain the relationship between built environment and travel behaviour [39,40], urban form and urban vitality [41,42], spatial analysis of urban areas [43,44]and so on. GBDT model takes into account non-linear relationships, are more complex than, for example, least squares regression, and tend to improve the interpretability of results [45]. GBDT regression model can be used to explore the impact of other geographical factors on the distribution of express delivery stations.

From the above review, it can be concluded that, with the development of information technology, the proportion of online shopping has increased year by year, which in turn has driven the development of related logistics and transportation as well as the growth of terminal logistics (express delivery stations). Driven by urban policies and new commercial service demands, express delivery stations have gradually clustered in urban communities, with population distribution, commercial facility distribution, and traffic accessibility all having an impact on this process. China’s current research mainly selects representative express pickup points, such as Cainiao Post and China Post, as research objects and focuses on their network layout and end distribution methods. The influencing factors on their distribution have only been initially explored in terms of economic development level, population distribution, and convenient transportation; relatively fewer studies have investigated the correlation between express delivery stations and other functional spaces in the city. This paper focuses on express delivery stations in Nanjing, including express self-pickup points and corporate express delivery stations, using GBDT model to explore the factors influencing their spatial distribution. All this leads to the discussion of the planning and management strategies of express delivery stations as community supporting facilities.

## Materials and methods

### Research area

**Fig 2:**
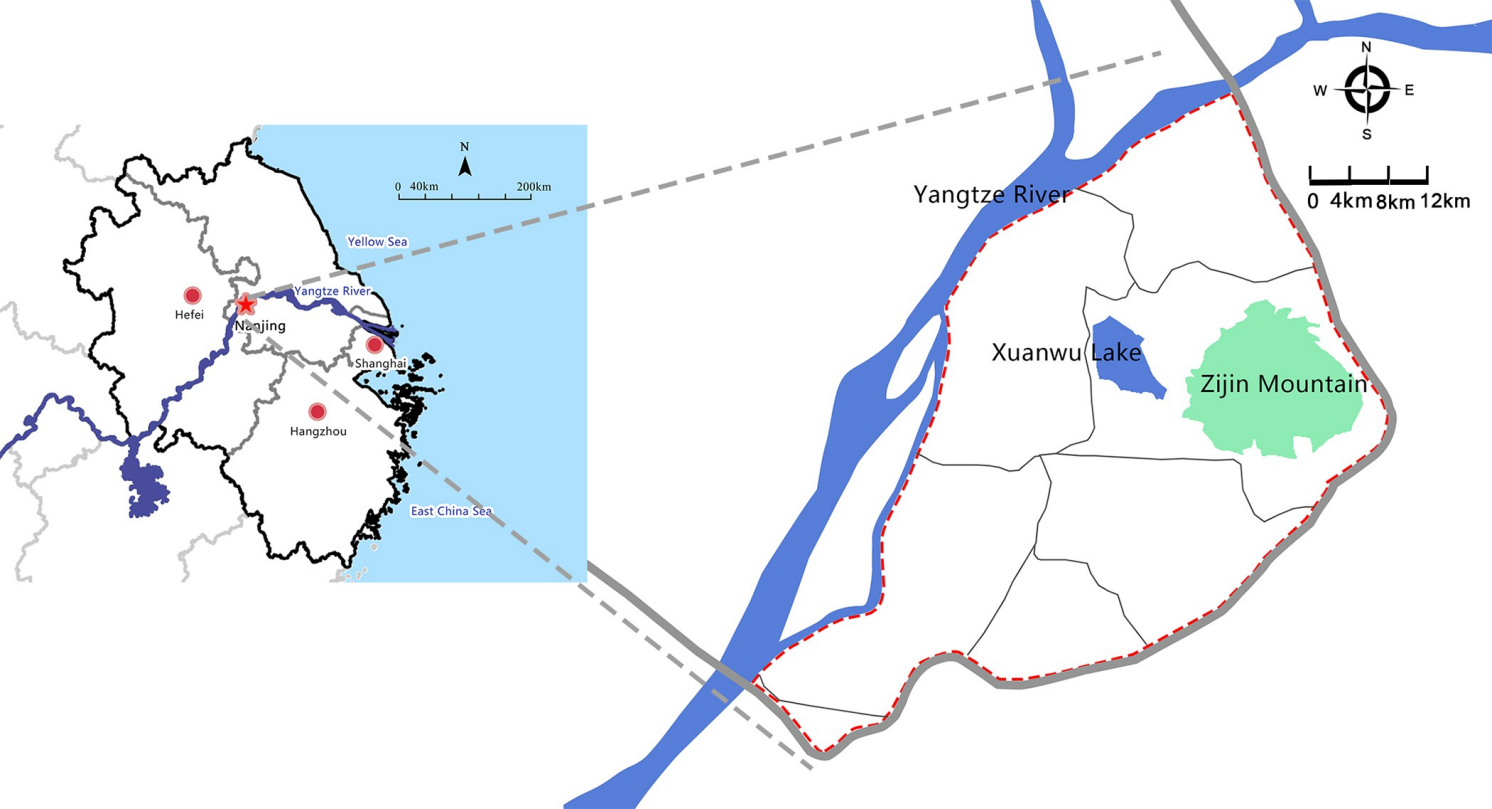
Location of Nanjing, China.

Nanjing, which is located in the Yangtze River Delta in eastern China, is the capital of Jiangsu Province. This is the only *Baoyouqu* area in China (i.e., the express cost of online shopping is borne by the seller) and is representative as one of the most developed areas in China’s online shopping market with prosperous online consumption. In this paper, the study area is delineated by the boundary between the City Ring Expressway and the Yangtze River, which is the central area of Nanjing (Fig 2).

### Data

POI (Point of Interest) data is used in this paper. POI abstracts different elements in the physical space into points with longitude and latitude coordinates and attribute labels. The POI data in this paper comes from the open-source data provided by the Amap platform. The data of express delivery stations from 2015 to 2021 were obtained for this paper. Express delivery stations in the AutoNavi Map are in the category of “life service; delivery; logistics”. We filtered the data by eliminating keywords containing “company,” "management center," "warehouse," "transport," "international transport," and other words of the data. According to the field-sampling inspection, the self-pickup points and collecting agency points of the major express companies are included by "logistics express," and the data is reliable. The data are mainly composed of five types of attributes: name, address (descriptive statement), latitude and longitude, administrative division, and category. A total of 451 pieces of POI data of express delivery sites were obtained in 2015; 1,404 pieces in 2016; 1,666 pieces in 2017; 1,826 pieces in 2018; and 2,071 pieces in 2019, which showed an increasing trend year by year. In 2020, there was a slight decrease due to the COVID-19 pandemic, and 1,964 data were obtained. In 2021, A total of 2,279 pieces of POI data of express delivery sites were obtained.

**Fig 3:**
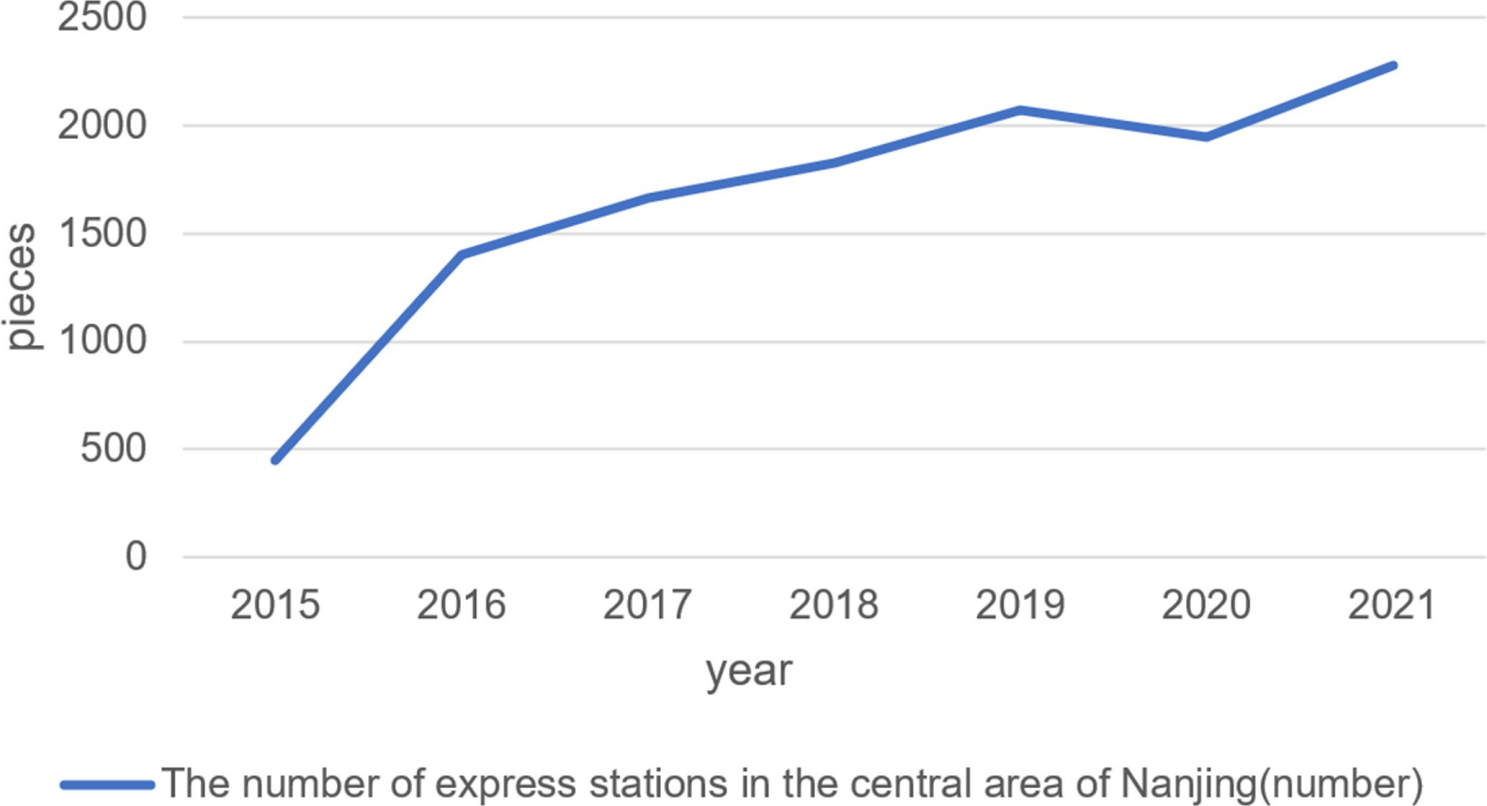
Changes in the number of express delivery sites in Nanjing from 2015 to 2021.

The online statistics of Nanjing Bureau of Statistics show that from 2015 to 2020, Nanjing’s online retail sales increased from 21.72 to 74.24 billion RMB, the express business volume increased rapidly from 502.519 to 951.0986 million pieces, which continuously improved in the next few years. In the same year, Jiangsu Provincial Government proposed to a professional service platform for the integrated development of logistics and acceleration of the improvement of the "last kilometer" terminal distribution network in the city. Starting in 2016, intelligent express delivery facilities such as Cainiao Station and Honeycomb began to spread across the country, and the number of express delivery stations in the central area of Nanjing showed a growth trend from 2015 to 2021 (Fig 3).

The agglomeration of express delivery stations has been increasing. The average nearest neighbor analysis of express delivery sites in Nanjing from 2015 to 2021 is shown in Table 1. The P values within 7 years are all less than 0.01, indicating that the clustering pattern is randomly generated each year with zero possibility. Additionally, the nearest neighbor index of express delivery sites in Nanjing is all less than 1, and there is a trend that the meanest observed distance value gradually decreases, indicating that their clustering degree in space progressively increases.

**Table 1:** Average nearest neighbor analysis results.

	Observation mean distance	Expected mean distance	Nearest Neighbor index	Z	P
2015	229.886370	438.229967	0.524579	-19.315112	0.000000
2016	131.846771	252.362701	0.522450	-34.232114	0.000000
2017	109.468294	231.292000	0.473290	-41.128188	0.000000
2018	101.965387	218.253588	0.467188	-43.556756	0.000000
2019	97.381565	206.938999	0.470581	-46.091457	0.000000
2020	108.435264	213.509368	0.507871	-41.531829	0.000000
2021	93.090941	196.7688140	0.473098	-48.120801	0.000000

The independent variables may affect the distribution of express delivery stations and were selected in the following ways. First, the distribution of express delivery stations may be affected by user demand [46], which can be divided into two parts: "shipping demand" and "receiving demand". Express delivery sites usually provide sending and receiving services for residential areas, enterprises, universities, scenic spots, businesses, etc. Second, the overall layout of express delivery stations in the central area of Nanjing also showed the characteristics of clustering in concentration in commercial centers. Therefore, commercial facilities, may affect express delivery station locations. It is mainly related to social retail, and other commercial behaviors as well as residents’ lives. According to the *Classification of Industry in National Economy (GB/T 4754–2017)*, this study classifies commerce into two types: daily retail and non-daily retail. "Receiving demand" is related to the places where residents live and work for a long time, such as residential communities, convenience stores and office buildings. Third, as one of the links of logistics, the distribution of express delivery stations is likely to be affected by traffic accessibility. Based on the literature review and the actual situation of Nanjing, this study selected seven variables from traffic, commercial, and population as three types of influencing factors of the spatial distribution of express delivery stations, which are described in. Specifically, this paper analyzes the influencing factors of express delivery stations in the central area of Nanjing in 2021, these POI data are also obtained from the open platform of Amap (Table 2).

**Table 2:** Selection of independent variable selection.

Type	Variable	Variable name	Description of Variable	Number
Population factors	X1	Residential areas	Refers to the large residential buildings with the relatively independent living environment in a certain area of the city	3667
	X2	Colleges and universities	Institutions of higher learning	864
	X3	Scenic spots	Tourist attractions	2597
	X4	Office	A commercial office building in a city or industrial park	1460
Commercial factors	X5	Daily retail	According to the Classification of Industries of National Economy (GB/T 4754–2017), general retail (521), specialized retail of food, beverage, and tobacco products (522) are classified into daily retail	8314
	X6	Non-daily retail	According to the Classification of Industries of National Economy (GB/T 4754–2017), the sales of automobiles, motorcycles, spare parts, fuel, and other power (526), the specialized retail of household appliances and electronic products (527), and the specialized retail of hardware, furniture and interior decoration materials (528) are classified as non-routine retail	13955
Traffic factors	X7	Bus and subway	Bus and subway stations	1661

## Methods

**Fig 4:**
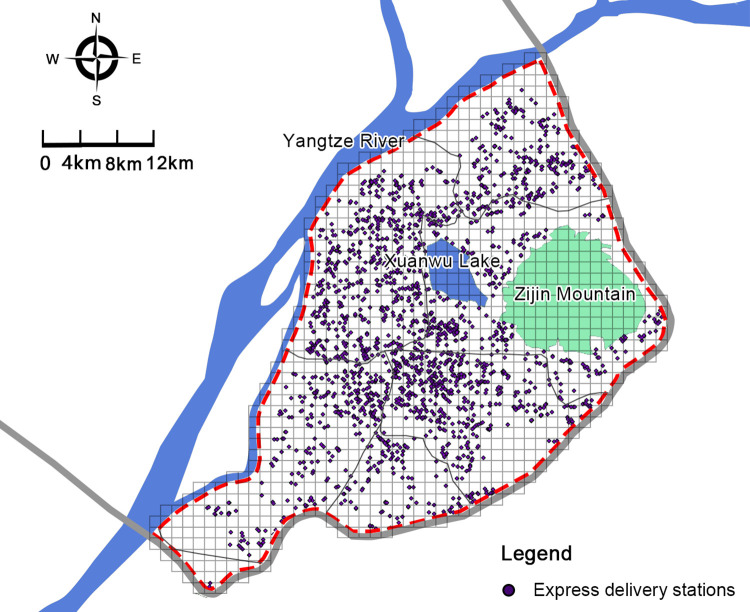
Distribution map of express delivery stations in the central area of Nanjing in 2021.

The grid measurement method was used to quantify the spatial distribution of the research data. According to the *Community Commercial Service Facilities Set and Functional Requirements (GB/T 37915–2019)*, express delivery station services need to cover neighborhoods with a service radius being smaller than 500 meters. In order to analyze the influencing factors of the spatial distribution of express delivery stations more conveniently, this study divided the research scope (the central area of Nanjing) into 3325 grids with a size of 500m*500m each (Fig 4). The number of each express delivery stations and the independent variable factors are counted in the statistical grid.

Then, the independent and dependent variables were substituted into more than a dozen machine learning algorithm models, from which the gradient boosting decision trees (GBDT) model with the highest R2 value was selected (Table 3) as the model for analyzing the factors influencing the distribution of express delivery sites.

**Table 3:** Calculation results of commonly used machine learning algorithm models.

	MSE	RMSE	MAE	MAPE	R^2^
Gradient Boosting Decision Trees	1.141	1.189	0.599	136.624	0.55
Decision Tree	4.41	2.1	1.463	51.823	0.102
Random Forest Regression	3.236	1.799	1.352	43.415	0.341
Adaboost	3.88	1.97	1.443	48.367	0.21
Extra trees	3.078	1.754	1.315	41.641	0.373
Catboost	3.382	1.839	1.405	44.099	0.311
KNN	3.215	1.793	1.379	45.598	0.345
Bp	3.096	1.759	1.318	41.655	0.369
SVR	358.533	18.935	16.29	509.758	-72.027
XGboost	4.406	2.099	1.583	53.937	0.103
LightGBM	3.927	1.982	1.472	47.249	0.2

Compared to other regression models, the GBDT has several advantages. First, it has the flexibility to handle various types of data, including continuous and discrete values. For example, the number of express delivery sites and other independent variables in the spatial grid are discrete variables that do not need to be converted to continuous variables. Secondly, it can accommodate missing data in independent variables whereas list-wise deletion is commonly adopted in regression models, which means that the number of independent variables in the grid can be zero. Thirdly, it can minimize the error and improve the accuracy of the model by a strong loss function, the final GBDT model can be viewed as a combination of many gradient regression trees. Many studies show that GBDT outperforms regression models.

The GBDT algorithm is an iterative decision tree algorithm, which can be viewed as an additive model consisting of *M* trees, with the following corresponding Eq (1). Assuming that *x* is a set of explanatory variables (i.e., the number of other geospatial elements in the grid) and *F*(*x*) is an approximate function of the response variable y (i.e., the number of delivery sites), the method estimates the function *F*(*x*) as an additive expansion *f*(*x*) based on the basis function h (x; w_m_); *h* is the categorical regression tree; *α* is the weight of each tree [47].


$$F(x,w)=\sum_{m=0}^{M}\alpha_{m}h_{m}\left(x;w_{m}\right)=\sum_{m=0}^{M}f_{m}(x,w_{m})$$ (1)

Given a training data set: $T=\{(x_{1},y_{1}),(x_{2},y_{2}),\ldots ,(x_{N},y_{N})\}$, *x*_*i*_∈*X*⊆*R*^*n*^, *X* for the input space, *y*_*i*_∈*Y*⊆*R*, *Y* for the output space, and a loss function of *L*(*y*,*f*(*x*)), our goal is to obtain the final regression tree *F*_*M*_.

(1) Initialize the first weak learner *F*_0_(*x*)

$$F_{0}(x)=\underset{c}{\arg\;\min}\sum_{i=1}^{N}L(y_{i},c)$$ (2)

(2) For building an M-tree classification regression tree *m* = 1,2,…,*M*
(a) For *i* = 1,2,…,*N*, calculate the response value (negative gradient of the loss function, i.e. pseudo-residual)

$$r_{m,i}=-{\left[\frac{\partial L(y_{i},F(x_{i}))}{\partial F(x)}\right]}_{F(x)=F_{m-1}(x)}$$ (3)

(b) For *i* = 1,2,…,*N*, fit data (*x*_*i*_,*r*_*m*,*i*_) using the CART regression tree to obtain *m* regression tree with a corresponding leaf node region of *R*_*m*,*j*_, where *j* = 1,2,…,*J*_*m*_, and *J*_*m*_ is the number of leaf nodes of the *m* regression tree.
(c) For *J*_*m*_ leaf node region *j* = 1,2,…,*J*_*m*_, calculate the best-fit value of

$$c_{m,j}=\underset{c}{\arg\;\min}\sum_{x_{i}\in R_{m,j}}L(y_{i},F_{m-1}(x_{i})+c)$$ (4)

(d) Update the strong learner *F*_*m*_(*x*)

$$F_{m}(x)=F_{m-1}(x)+\sum_{j=1}^{J_{m}}c_{m,j}I(x\in R_{m,j})$$ (5)

(3) Obtain the expression for the strong learner *F*_*M*_(*x*)

$$F_{M}(x)=F_{0}(x)+\sum_{m=1}^{M}\sum_{j=1}^{J_{m}}c_{m,j}I(x\in R_{m,j})$$ (6)

GBDT is capable of handling discontinuous independent variables and fitting complex non-linear relationships of independent variables [48]. It quantifies the relative importance or contribution of each independent variable in predicting the response and identifies and ranks the influence of the independent variables on the response prediction. For a single decision tree T, Breiman et al. [49] proposed the following metric as an approximation of the relative importance of the predictor variable x in predicting the response:

$$I_{\kappa }^{2}(T)=\sum_{t=1}^{J-1}\overset{\wedge }{\tau_{t}^{2}}I(v(t)=\kappa )$$ (7)

where the summation is over the non-terminal nodes *t* of *J*-terminal node tree T, *x*_*κ*_ is the splitting variable associated with node *t*, and $\hat{\tau_{t}^{2}}$ is the corresponding empirical improvement in squared error as a result of using predictor *x*_*κ*_ as a splitting variable as the non-terminal node t. For the set of decision trees ${\left\{T_{m}\right\}}_{1}^{M}$, obtained by the gradient boosting method, Eq (7) can be generalized by its average over all additive trees as follows [45]:

$$I_{\kappa }^{2}=\frac{1}{M}\sum_{m=1}^{M}I_{\kappa }^{2}(T_{m})$$ (8)

This study applied the "Gradient Boosting Regressor" package from sklearn in python GBDT regression model to calculate the data for 2021. Specifically, the samples were divided into ten subsets, and the model was fitted using seven different subsets (70% of the data) and validated by the remaining subsets (30% of the data). Three important parameters need to be determined: the number of trees, the learning rate, and the depth of the trees. The model learning rate was fixed at 0.001, at which the prediction bias of the model was low. Also, the number of trees was set to a maximum of 10,000. A series of tests were carried out on the interaction depth level of the trees to obtain the best results. The final model was based on a tree complexity of 10. Finally, the best model had a pseudo-R2 of 0.55 and then the relative importance of the independent variables was derived for further analysis.

## Results

According to the results of the gradient boosting decision trees regression analysis, the relative contributions of independent variables were shown in Table 4 and the contribution is measured in a relative way so that their total contributions amount to 100%.

**Table 4:** Influence level of independent variables.

Type	Variable	Variable name	Metro ridership	
			Rank	Relative importance (%)
Population factors	X1	Residential areas	2	15
	X2	Colleges and universities	7	1
	X3	Scenic spots	6	3
	X4	Office buildings	4	6
Commercial factors	X5	Daily retail	1	59
	X6	Non-daily retail	3	11
Traffic factors	X7	Bus and subway	4	4

We ranked independent variables according to the size of their relative importance. Daily retail is the most important variable for the distribution of express delivery stations, with a 59% contribution. followed by residential areas (15%) and non-daily retail (11%). The results show that office buildings (6%), public transport facilities (4%) scenic spots (3%) and universities (1%) have a weak influence on the distribution of express delivery stations. It can be seen that commercial facilities have the greatest influence on the distribution of express delivery stations. Among the Population factors, only the residential areas and office buildings have important influence on the distribution of express delivery stations, while the other factors have little influence on it. In addition, the traffic factors do not have a significant impact on it.

The results show that commercial and residential factors have a strong impact on the distribution of express delivery stations. With the continuous development of e-commerce in recent years, great changes have taken place in the market environment of social retail. Physical stores often carry out an integrated online and offline business model. Besides, express delivery stations are often set up together with daily retailing such as supermarkets, convenience stores, and food markets as well as other shopping locations that are closely related to residents’ daily lives. Therefore, the correlation between daily retail and express delivery stations is the most significant.

Non-daily retail also has a significant relationship with the layout of express delivery stations. Self-service transportation of non-daily retail after offline purchase is difficult to a certain extent, and express delivery has become the choice of people. Studies have shown that the layout of express delivery stations is related to the industries in the region [50]. The Zhujianglu area is a concentration of the electronics industry in Nanjing, with customers from all over the country and a high demand for express delivery services, so the distribution of express delivery stations is more concentrated. The clustering of express delivery stations around Xianlin Auto City, where the automobile supporting industry is concentrated, has also become more prevalent, verifying the significant impact of non-daily retailing on the layout of express delivery stations.

In the functional areas where the population is concentrated, the influence of residential areas on the distribution of express delivery stations is also more significant, while office buildings, scenic spots, universities and public transport facilities have a weak influence on the distribution of express delivery stations. Tan et al. (2016) surveyed residents’ self-pickup behavior in Nanjing and concluded that compared with residential areas, employees working in office buildings in commercial areas are less willing to use express delivery self-pickup space [17]. The work of the Hexi CBD office, Xinjiekou office, and Xuzhuang industrial park office research showed that most of the couriers offer door-to-door delivery for office buildings, and that 80% of the employees were more willing to accept door-to-door delivery and collection services, with only the remaining 20% willing to go to pick-up express delivery stations during their break time, which also verifies that there is a weak spatial correlation between express delivery stations and office buildings.

## Discussion

This paper combines the method of spatial grid counting with machine learning model methods to provide new ideas for spatial analysis. We have applied the GBDT model to the study of influencing factors of express delivery stations’ spatial distribution, effectively avoiding the problems encountered in the use of traditional regression models in spatial analysis, such as the discontinuity of the independent variables and the lack of accuracy of the results. There are also some limitations in this study. Firstly, The POI data of a single platform was used in this study, and the accuracy of the data still needs to be cross-verified. Secondly, the GBDT method cannot generate confidence intervals for the predictors. It can be speculated that variables with weak relative importance are statistically insignificant. However, we do not know the threshold of "weakness". Therefore, we used relativity to explain the effect of each variable on the layout of express sites.

**Fig 5:**
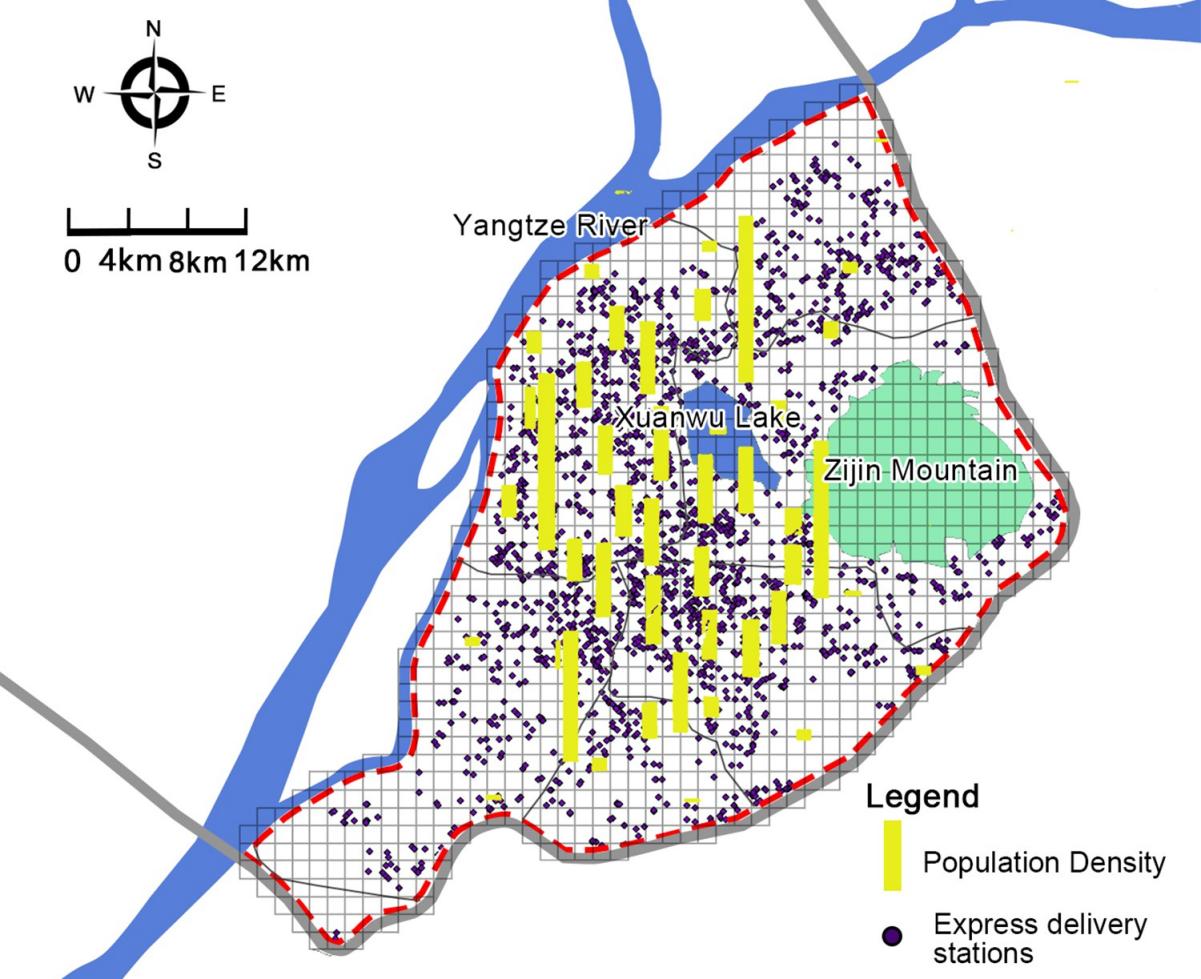
Relationship between express delivery stations and population density.

Many studies have examined the pattern of distribution of express delivery stations in space. Express delivery stations are mostly distributed in densely populated areas close to customers [51]. Some studies in China have considered communities and enterprises as important factors affecting the distribution of express delivery stations [26,27], In our study, it can be seen that residential areas have the largest impact on the distribution of express delivery stations. As seen in Fig 5, Nanjing’s old city has dense residential areas, high population density, and dense distribution of express delivery stations.

Some studies believe that university and college students are an important group in online shopping, and that there is a correlation between colleges and express delivery stations in terms of layout [52,53]. However, the results of this study show that colleges and universities have no significant influence on the distribution of express delivery stations because of the particularity of land use of colleges and universities, which covers a large area and has a small total number of colleges and universities, making it difficult to form agglomeration. In addition, most colleges and universities do not set up express delivery stations internally, and college express delivery stations are usually set up in combination with the surrounding residential areas and stores, which further weakens this correlation.

Studies in France and other countries show that the distribution of express delivery stations and commercial spaces are closely related [32,35], while in Chinese studies, more attention is usually paid to the relationship between express delivery stations and living areas such as residential areas and universities [17]. The case of this study fully demonstrates the trend of spontaneous construction of express site space to commercial retail clustering, and also proves that the arrangement of express delivery stations combined with customers’ daily consumption places is an inevitable product of the market role. At the same time, this paper also finds that the distribution of express sites and bulk items also has a strong correlation, which is mainly related to the demand for consignments.

The proximity of express delivery stations to bus and subway stations is, to some extent, conducive to improving the efficiency of picking up items for customers. Studies from developed countries show that express delivery stations and rail way stations are inextricably linked [35]. For example, Pickup, the express network of France Post, has set up express cabinets in subway and city railway stations to facilitate commuters to receive express deliveries. However, our research shows that Nanjing’s express delivery stations are less relevant to public transportation, mainly because people usually pick up their deliveries on foot and do not rely on public transportation, and the high rents around bus and subway stations is not conducive to the location of delivery stations. In addition, express deliveries are usually delivered to the households in China and therefore express delivery stations are more often to be arranged in conjunction with community commercial facilities and community amenities, making it more convenient for residents to access their packages.

## Conclusions

This study explores the relationship between express delivery stations and commercial facility factors, population factors, and transportation factors in Nanjing, China, in 2021. We used the GBDT model to quantify the relative importance of other geospatial factors on the distribution of express delivery stations, complementing the application of the GBDT model in urban studies. As far as we know, there are few studies that apply the GBDT model to spatial distribution influencing factors. It provides insightful results to the literature.

The results of this study have important implications for rational urban planning. The Internet has had a significant impact on the shopping behavior of residents and has given rise to the emergence of new commercial spaces such as express delivery stations. The existing commercial service support system lacks an online component, which is also reflected in the configuration of express delivery stations. How to cope with the rapid development of express delivery stations has become a new challenge for city managers and planners. As mentioned above, the express delivery station space is closely related to the daily retail space of the community (such as supermarkets, convenience store, food markets). The mode of cooperation between community retail and express delivery enterprises undoubtedly increases their attractiveness to customers and at the same time, facilitates the daily shopping needs of customers. Most of non-daily retail is located in the commercial block, which further proves that the express delivery site has the tendency of clustering to the consumption space. In addition to all this, the residential area has been determined to be another major factor affecting the distribution of express delivery stations. Compared with offices, scenic spots, and colleges, residential areas are the most important places for people’s daily lives and the urban functional areas with the greatest and most stable demand for delivery stations. However, there are certain irrationalities in the current layout of express delivery stations under the role of the market, such as insufficient support in the community; failure to combine with residents’ commuting routes, etc., which should be considered in later planning. Therefore, this study will help urban planners to better plan the layout of express delivery stations, so as to improve the rationality of the spatial distribution of express delivery stations in the city.

Limited by data sources, this paper selects only spatial elements that can count quantitative points in the selection of influencing factors and does not include economic and consumer behavior factors, which should be included in subsequent studies.
